# Risk communication and community engagement as an emerging pillar of health emergency management in Iran: Achievements and the way forward

**DOI:** 10.3389/fpubh.2023.1097932

**Published:** 2023-02-15

**Authors:** Mikiko Senga, Marzieh Kouhestani, Sayed Mohsen Hosseini Boroujeni, Ebrahim Ghaderi, Peyman Parchami, Syed Jaffar Hussain

**Affiliations:** ^1^WHO Health Emergencies Programme, World Health Organization, Geneva, Switzerland; ^2^WHO Health Emergencies Programme, World Health Organization, Tehran, Iran; ^3^Department of Communication Science, Faculty of Communication Science, Allameh Tabatabai University, Tehran, Iran; ^4^Health Policy Research Center, Institute of Health, Shiraz University of Medical Sciences, Shiraz, Fars, Iran; ^5^Center for Communicable Diseases Control, Ministry of Health and Medical Education, Tehran, Iran

**Keywords:** risk communication, community engagement, COVID-19, health hazards, infodemics

## Abstract

This article is part of the Research Topic Health Systems Recovery in the Context of COVID-19 and Protracted Conflict.

Risk communication and community engagement (RCCE) is an essential component of emergency preparedness and response. In Iran, RCCE is a relatively new area of public health. During the COVID-19 pandemic in Iran, the national task force relied on conventional methods, which is to utilize existing primary health care (PHC) structure to implement RCCE activities around the country. The PHC network and the community health volunteers embedded in it enabled the country to bridge the health system and communities from the very beginning of the COVID-19 pandemic. The RCCE strategy to respond to COVID-19 was adapted over time with the development of a national program, commonly known as the “Shahid Qassem Soleimani” project. This project consisted of six steps including case detection, laboratory testing through the establishment of sampling centers, scale up of clinical care to vulnerable groups, contact tracing, home care for vulnerable population, and COVID-19 vaccination roll out. Nearly 3 years into the pandemic, the importance of designing RCCE for all types of emergencies, allocating a dedicated team to RCCE, coordinating with different stakeholders, improving the capacity of RCCE focal points, practicing more efficient social listening, and using social insight for better planning were identified as some lessons learned. Further, Iran's RCCE experience during the COVID-19 pandemic underscores the importance of continuing to invest in the health system, particularly PHC.

## Introduction

Coronavirus disease 2019 (COVID-19) outbreak took the world by surprise, as SARS-CoV-2 virus rapidly spread from Wuhan, China to 114 countries giving rise to more than 118,000 confirmed cases and 4,291 deaths by the time the outbreak was declared a pandemic by the World Health Organization on 11 March 2020 (1). Soon thereafter, countries around the world started closing their borders, and public health officials urged people to wear masks, social distance, and practice hand hygiene (2). As people were urged to stay home amidst increasing uncertainty of the pandemic's trajectory, the pandemic heightened their anxiety.

The COVID-19 situation in the Islamic Republic of Iran was no exception. The first case in the country was a 68-year-old man, who was admitted to a hospital on 12 February 2020 in Qom, a holy city that welcomes thousands of tourists every year. He tested positive for COVID-19 on 19 February 2020, along with six additional confirmed cases from the same city. All seven confirmed cases lost their lives as of 23 February 2020 (3). The virus rapidly spread to neighboring areas, such as Tehran, Markazi, Isfahan, and Semnan provinces (4).

**Figure 1:**
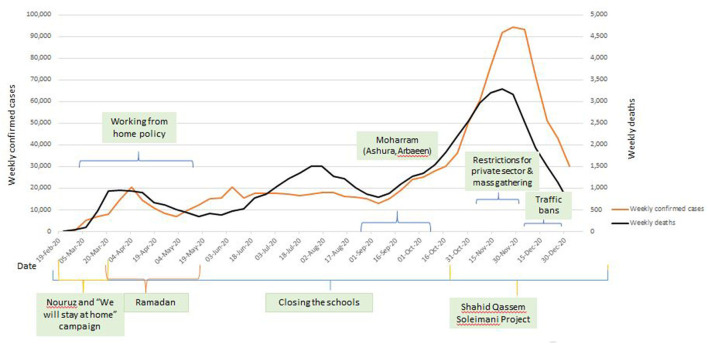
COVID-19 epidemic curve with public health and social measures implemented in 2020.

Between 19 February to 16 March 2020, Iran had the highest number of confirmed cases after China and Italy and was the hardest hit country in the WHO's Eastern Mediterranean region, with 14,991 cases and 853 deaths (4). A national task force for the COVID-19 response was formed under the President's office, and the Ministry of Health and Medical Education (MoHME) took the technical lead in the task force. As of January 2023, this task force continues to have the highest authority in the management of COVID-19. It serves as a coordination forum and advises on intersectoral collaboration and coordination, policy making, and monitoring and evaluation of outbreak control strategies, including surveillance, epidemiological investigation, contact tracing, points of entry, laboratory detection, infection prevention and control, case management, and vaccination. The task force also put in place a range of nonpharmaceutical public health and social measures (PHSM) to contain the virus spread around the country, such as closure of public places including schools and universities, travel ban, reduced working hours, COVID-19 hotlines, online screening platforms, and awareness raising campaigns (Figure 1) (5). Risk communication and community engagement (RCCE) emerged as an essential component of these aspects of the COVID-19 response.

According to the World Health Organization, risk communication is “the real-time exchange of information, advice and opinions between experts or officials and people who face a threat (hazard) to their survival, health or economic or social wellbeing” (6). Further, community engagement refers to “a process of developing and motivating relationships that enable stakeholders to work together to address health-related issues and promote wellbeing to achieve positive health impact and outcomes” (7). In its ideal form, RCCE is a proactive, two-way and iterative interaction between public health professionals and affected populations concerning a health-related hazard, with an intent to build trust to maximize appropriate prevention and control behaviors and actions in a health emergency. RCCE empowers individuals from the affected populations to make informed decisions, not only to protect themselves from the hazard but also to contribute to improving the health of others in their communities. At the same time, it enables public health professionals to establish effective means to protect the health of the population.

The use of RCCE as a guiding tool in emergency response is relatively new although elements of RCCE, particularly one-way risk communication to communities, have existed for decades. The field has evolved from risk communication, which is one-way provision of health information to communities, to risk communication and community engagement, which involves two-way communication to encourage participation by people in affected communities to co-create and disseminate knowledge and information. RCCE has direct benefits in mitigating health-related risks in disasters (8, 9), as it can play a role in managing mis- and dis-information and infodemics (10, 11), and in maximizing local capacities to shape an emergency response (12, 13). In Iran, the health workforce has increasingly recognized the significance of community engagement and resilience in disaster and emergency preparedness (14). RCCE in the early phase of the COVID-19 outbreak relied on conventional methods that have existed in the country since 1985, the year when the foundations of primary health care (PHC) in Iran were laid. As the pandemic evolved, the health authorities began to acknowledge the importance of listening to communities to enable RCCE activities and also significantly expanded the range, reach, and intensity of two-way communication.

Recognizing the continued importance of RCCE in future emergency preparedness and response, we reflect on the COVID-19 response in the country, in particular, to describe conventional RCCE methods that were employed in the initial public health response, ways in which the RCCE strategies adapted over the course of the outbreak, and a future direction based on the lessons learned.

## Risk communication and community engagement approaches at the onset of COVID-19 outbreak

### Pre-existing structure of primary health care and community health volunteering in Iran

PHC in Iran has a robust network, consisting of four main types of primary health facilities:

1) Health houses serve rural areas with their locations depending on the geography of the catchment area. They are staffed by “Behvarz”, who have received a two-year certified training, and deliver health services to approximately 700 people;
2) Rural comprehensive health centers cover five health houses or approximately 7,500 people;
3) Health posts in urban areas provide care to 2,500 people, which are supported by family health care providers known as “Moragheban-e-salamat”, who enter the workforce with bachelor's level health-related education; and
4) Urban comprehensive health centers which oversee the day-to-day operation of three health posts provide healthcare to roughly 30,000 population.

Within this PHC structure, there are community health volunteers who are directly and regularly trained and supervised by the four types of health facilities. These volunteers are known as “Rabetan-e-salamat” and “Safiran-e-salamat”, and they both provide health education and personalized follow up for medical or health related issues. The former is assigned to a neighborhood, which is the catchment area, whereas the latter serves in an “ambassador” role to obtain knowledge from the health facilities and transfers it back to her own extended family (15).

### Leveraging the pre-existing capacity of PHC and community health volunteers during early stages of the pandemic

PHC serves as the first point of contact of all individuals who are seeking healthcare, and thus provides a structural foundation of effectively responding to health emergencies (16). In this system, community health workers (CHWs) are provided with sufficient training on how to interface with members of their community to transfer knowledge from health authorities and listen to concerns raised by communities. Concerns or questions are then brought back to the health authorities to be addressed or responded to, and the feedback loop continues. In parallel, the community members spread the knowledge gained to their peer groups.

Given PHC's routine scope of work that includes health education, public health awareness raising campaigns, health promotion, community and stakeholder engagement, one of the most recognized community engagement activities in response to COVID-19 was leveraging the capacity of existing community health volunteers in PHC. They were actively involved in communication with suspected, probable, and confirmed cases, providing them with health information regarding when and how to visit their physicians and how to prevent transmission to others. In addition, the volunteers played a role in contact tracing by obtaining information regarding contacts from the cases and monitoring the health condition of the identified contacts. Since the onset of COVID-19, Behvarz and Moragheban-e-salamat have received additional courses on COVID-19 to improve their knowledge and practical skills.

Both types of community health volunteers (Rabetan-e-salamat and Safiran-e-salamat) supported other members of the community to participate in co-creating the information and message transfer to all target groups. For example, the Ministry of Health continuously provided up-to-date COVID-19 information to CHWs, who repackaged it in a culturally appropriate manner for their target audiences. Likewise, the information was made accessible to those with special needs or disabilities. For example, Behvarz disseminated COVID-19 related messages orally to those who were illiterate or blind. The volunteer system in the country was able to reach a wide range of populations, which also included people living with addiction, pregnant women, people living in remote areas, and refugees.

## Adapting the risk communication and community engagement structure to respond to COVID-19

The national COVID-19 task force developed a program for the prevention and control of COVID-19, commonly known as the “Shahid Qassem Soleimani” project, named after a late military official, based on national priorities. Six priorities were implemented in a stepwise approach, with each step building on preceding step(s) (Table 1) while RCCE strategies and activities were adapted to suit the objectives of each step or priority.

**Table 1:** Six steps of the national COVID-19 response project.

1- Screening and case detection of the catchment population through phone interview by community health volunteers, hotline, and self-reporting platforms
2- National mobilization of laboratory screening through the establishment of sampling centers in selected COVID-19 health facilities
3- National mobilization to scale up clinical care to those who could not receive necessary health care services
4- Conduct contact tracing, home care for vulnerable population, and neighborhood care through the support of the Iranian Red Crescent Society and local voluntary organizations
5- National mobilization for COVID-19 vaccination roll out
6- Encourage maximum community engagement in response to COVID-19 for vaccination, public health and social measures, and collaboration coordination of all governmental and non-governmental organizations, institutes, and offices using new technologies

In the first step, the screening of individuals was conducted for active case finding purposes. The community health volunteers called by telephone suspected and probable cases and associated contacts in their catchment area to monitor symptoms that would meet the COVID-19 case definition (17). Those who had symptoms were promptly referred for testing and/or clinical care. The community health volunteers also gathered data of suspected cases and entered the data either into a dedicated online surveillance database of the MoHME or delivered the data to Moragheban-e-salamat to be compiled into the integrated health platform.

The second step was aimed at expanding sampling and testing of suspected cases to break the chain of transmission. The community health volunteers, along with other governmental and non-governmental organizations, conducted rapid assessment of self-reported suspected cases, people over 60 years old, individuals with underlying medical conditions, and other high-risk groups (e.g., pregnant women) for this purpose. Additionally, the volunteers were trained to assess high-risk contacts during their visits to houses and recommend laboratory testing as required, thereby contributing to contact tracing efforts in the community.

The third step was dedicated to ensuring provision of routine care to those with chronic conditions who were refused care due to COVID-19 related concerns. Those who were deemed to be at high risk yet were not receiving their routine medical care were identified and abstracted from an integrated health platform of the MoHME. They were subsequently called by CHWs and volunteers, not only screening them for COVID-19 but also inviting them to come to PHCs to receive their regular health services. Through this step, 95% of the at-risk population received telephone calls from community health volunteers (MoHME data).

The fourth step was devised to manage and control the COVID-19 pandemic with public participation and coordination between two departments of the MoHME, namely Education and Health Promotion and Public Relations, specifically in the areas of contact tracing, home care for vulnerable population, and neighborhood care. In this step, RCCE efforts were scaled up with the establishment of four sub-teams:

a) The surveillance-care team was responsible for active tracing of patients, following up *via* phone calls, performing the rapid test, and providing home care;
b) The monitoring team was responsible for supervision and monitoring the quality of implementation of health instructions in public places. Members of this team were recruited from Iranian voluntary organizations;
c) The support team was responsible for supporting the families of patients and people who were in home isolation or quarantine, as well as people who experienced economic losses due to the mandatory health protocols; and
d) The risk communication and community engagement team was responsible for educating patients and families, public education, and managing infodemics and rumors. Training topics included media literacy, quarantine, hygiene guidelines, and, in the fifth step, persuasive communication to maximize vaccination uptake. Persuading governmental and non-governmental organizations to provide health and educational services in a PHC context was also performed by this team.

The RCCE methods in the fourth step carried over to the fifth step, making RCCE critical in the nationwide vaccination roll out. In this step the RCCE task force ramped up RCCE efforts by utilizing the influential power of religious leaders, athletes, artists, and experts as a key strategy in awareness raising campaigns to minimize vaccine hesitancy. To further entice people to get vaccinated, a travel ban was introduced for those who have not been fully vaccinated. As of November 2022, 82% of the population received their first dose of a COVID-19 vaccine, and 69% are fully vaccinated (MoHME data).

In addition to RCCE activities that were implemented under the six priorities of the “Shahid Qassem Soleimani” project, both official and unofficial efforts were made to enhance public health response. As with any health emergency response, a spokesperson was appointed at the national level, and additional spokespersons were designated in each of 64 universities of medical sciences in the country. Many campaigns were organized virtually as well as in person with the spokesperson delivering messages that had been generated and agreed by the COVID-19 task force. Such campaigns raised awareness about COVID-19, offered advice on how to prevent and treat it, and stressed the importance of adhering to PHSMs. Moreover, a new national committee was formed to recommend a variety of other RCCE activities which were endorsed by the MoHME, Ministry of Education, Ministry of Culture and Islamic Guidance, and Ministry of Higher Education, among others. A notable example is social listening, which is intended to “track, analyze and synthesize community inputs both digitally and offline” (10). To enable it, existing hotlines of MoHME and social welfare organization were repurposed for COVID-19 response so that resources could be dedicated 24 hours, seven days a week to respond to questions, concerns and critical opinions of the public as well as to provide free counseling on psychosocial issues. More informally, public health response relied on Iran's charity network to secure and distribute masks, hand sanitizers, food, and other necessities to vulnerable communities (18).

## Lessons learnt and persistent challenges to RCCE during the response to COVID-19

While Iran's health system structure proved to be advantageous, COVID-19 certainly tested RCCE in this structure, as RCCE in Iran is not designed for all types of emergencies. Moreover, a dedicated unit or team specifically for RCCE did not exist in the government structure at the beginning of the pandemic. Consequently, there was limited coordination among different stakeholders which resulted in numerous duplications or incongruent activities (19). However, COVID-19 propelled the country to strengthen its RCCE structure. For example, the Education and Health Promotion Department of MoHME developed the RCCE national plan, in consultation with relevant stakeholders in various parts of the government, non-governmental organizations, and the media, to ensure multisectoral coordination and collaboration. Further, provincial universities have been involved in the implementation of RCCE activities to reach broad catchment areas and a variety of target groups.

At the beginning of the pandemic, RCCE focal points of the MoHME and in provinces did not have sufficient training and capacity to effectively and efficiently deliver RCCE interventions. For example, although media monitoring had been in place, it was not sufficiently organized to encourage community participation and community resilience. Moreover, while 73% of Iranian people followed COVID-19 news *via* social media as well as national mass media (20), there were insufficient mechanisms to capture community insight and track misinformation and dis-information. In addition, these insights were not monitored for outbreak response purposes, resulting in missed opportunities to generate and disseminate appropriate responses to concerned communities.

Another notable lesson learned is the need for operational research in the area of RCCE during outbreaks and other health emergencies. While many studies, such as knowledge attitude and practice surveys, were conducted both nationally and at the provincial level, there was insufficient emphasis on knowledge translation. Thus, study results were not applied in real time to inform the RCCE response and translate into policy. Using evidence and insights from communities to adjust strategies during an emergency response would reduce the duration of the emergency as well as morbidity and mortality of the affected population (21, 22). Similarly, studies aimed at understanding the feedback loop between health professionals and affected populations would provide insight on information flow, mediating factors, and actions taken, which would in turn strengthen two-way communication required for optimizing a health emergency response. The same could be said for the co-creation of information in community settings, which was anecdotally reported but not systematically documented. Studies to understand the capacity of communities and factors that drive community engagement would inform preparedness and response to public health emergencies in the future.

## Discussion

Owing to Iran's primary health care network and the community health volunteers embedded in it, the country has had a structure to connect the health system and communities from the very beginning of the COVID-19 pandemic, allowing the implementation of RCCE activities in a systematic manner. The pandemic reinforced RCCE as a critical component in health emergency preparedness and response, and now, 3 years since COVID-19 emerged, RCCE is included in all phases of emergency management (i.e., mitigation, preparedness, response, and recovery) in the country.

Due to its geographical location, Iran faces a number of potential hazards, including natural hazards (e.g., earthquakes), migration of Afghan refugees, and annual cross-border movement of people for mass gathering events, all of which carry a risk of outbreaks. Building on the experiences from COVID-19, Iran has already begun preparing for future health emergencies. Of note, the MoHME in partnership with Tabriz University of Medical Science conducted a workshop to develop a national preparedness and response plan for influenza and other respiratory viruses in July 2022, which will be followed by a simulation exercise. The MoHME also took the lead in hosting a “G5” meeting in September 2022, inviting the neighboring countries of Afghanistan, Iraq, and Pakistan, with the support of the World Health Organization, to strengthen cross-border collaboration to mitigate risks during health emergencies. To prepare for and respond to potential cross-border transmission of high threat pathogens during the pilgrimage of Arbaeen, which is the world's largest annual mass gathering, RCCE was activated to protect at least five million Iranians from health-related hazards, such as heat stroke, food-borne diseases, and traffic accidents, and even stampede and terrorist attacks.

To build on these achievements so that Iran's RCCE can flourish in the future, it is essential to strengthen multi-sectoral coordination, capacity of spokespersons, healthcare providers, community volunteers, and others who are working in RCCE, social listening techniques, community-led initiatives to engage community members, monitoring and evaluation, and reporting. Moreover, recognizing that the effectiveness of RCCE interventions is dependent on public trust and vice versa (23), there is a need to leverage the power of individuals in the community who people trust and those active in social media and to maintain a continuous dialogue between the governmental entities, humanitarian organizations, other actors in disaster management, and the public (24). Finally, improving the level of health literacy would complement RCCE, as it is associated with trust building (25, 26) and can ultimately affect patient outcomes due to health disparities (27).

In summary, Iran's RCCE experience during the COVID-19 pandemic underscores the importance of investing in the health system, particularly primary health care. Considering cultural factors of community engagement, the capacity of voluntary organization and community members are a significant asset in responding to a major health hazard. With unique challenges, such as the economic sanctions including foreign trade, financial services, and technologies, strengthening the capacity in all aspects of RCCE is essential for sustainability purposes, especially considering that certain tools, such as social listening platforms and fact checking services, are available internationally but not in Iran, hindering Iran's ability to fully implement RCCE. Promoting a fair and equitable response to COVID-19 has been extensively highlighted in this pandemic (28), and this guiding principle is perhaps the most important as the country continues to manage the COVID-19 pandemic while addressing health system recovery and resilience and preparing for future health emergencies.

## Data availability statement

The original contributions presented in the study are included in the article/supplementary material, further inquiries can be directed to the corresponding author.

## Author contributions

MK, MS, and SMHB contributed to the main ideas in collaboration with EG, PP, and SJH. MK wrote the first draft of the manuscript. MS as the senior author, critically reviewed, substantially revised, and polished the entire manuscript. All authors contributed to the manuscript review and revision and approved the final version.
